# Pro-Inflammatory Cytokines as Core Mediators of Colonic Epithelial Barrier Dysfunction: Roles of TNF-α, IFN-γ, IL-1β, and IL-6

**DOI:** 10.3390/ijms27114722

**Published:** 2026-05-24

**Authors:** Dinesh Prasad V Thanga Velu, Mh Busra Fauzi, Faizul Jaafar, Norfilza Mohd Mokhtar, Mohd Helmy Mokhtar, Adila A Hamid

**Affiliations:** 1 Department of Tissue Engineering and Regenerative Medicine, Faculty of Medicine, Universiti Kebangsaan Malaysia, Cheras 56000, Malaysia; p153533@siswa.ukm.edu.my (D.P.V.T.V.); fauzibusra@ukm.edu.my (M.B.F.); 2 Department of Biochemistry, Faculty of Medicine, Universiti Kebangsaan Malaysia, Cheras 56000, Malaysia; faizuljaafar@ukm.edu.my; 3 International Medical School, Management and Science University, Shah Alam 40100, Malaysia; norfilza@msu.edu.my; 4 Department of Physiology, Faculty of Medicine, Universiti Kebangsaan Malaysia, Cheras 56000, Malaysia; helmy@ukm.edu.my

**Keywords:** pro-inflammatory cytokines, colon barrier, barrier dysfunction

## Abstract

The colonic epithelial barrier is a multilayered defense system comprising the mucus layer, intestinal epithelial cells (IECs), and the underlying lamina propria. These components collectively maintain mucosal homeostasis and restrict microbial translocation. Disruption of this barrier is a hallmark of chronic intestinal inflammation particularly in IBDs, and is primarily driven by pro-inflammatory cytokines, such as TNF-α, IFN-γ, IL-1β, and IL-6. TNF-α and IFN-γ synergistically induce epithelial cell apoptosis and tight junction disassembly through mechanisms involving TNFR2 upregulation, myosin light chain kinase (MLCK) activation, and adherens junction destabilization. IL-1β amplifies paracellular permeability via NF-κB-dependent MLCK induction and OCLN downregulation, while IL-6 promotes barrier leakiness by upregulating CLDN-2 and sustaining self-reinforcing inflammatory loops that maintain chronic inflammation and impede epithelial repair. This leads to persistent immune-cell infiltration, chronic tight junction remodeling, and failure of barrier replenishment. Consequently, leaky colon facilitates microbial and antigen translocation into the lamina propria, further activating immune cells and perpetuating pro-inflammatory signaling. This review synthesizes current evidence and studies on the cooperative and self-reinforcing roles of pro-inflammatory cytokines, providing insight into the mechanisms underlying chronic intestinal barrier dysfunction and highlighting the need for therapeutic strategies that simultaneously target multiple inflammatory axes to restore barrier integrity in inflammatory bowel disorders.

## 1. Introduction

**Figure 1:**
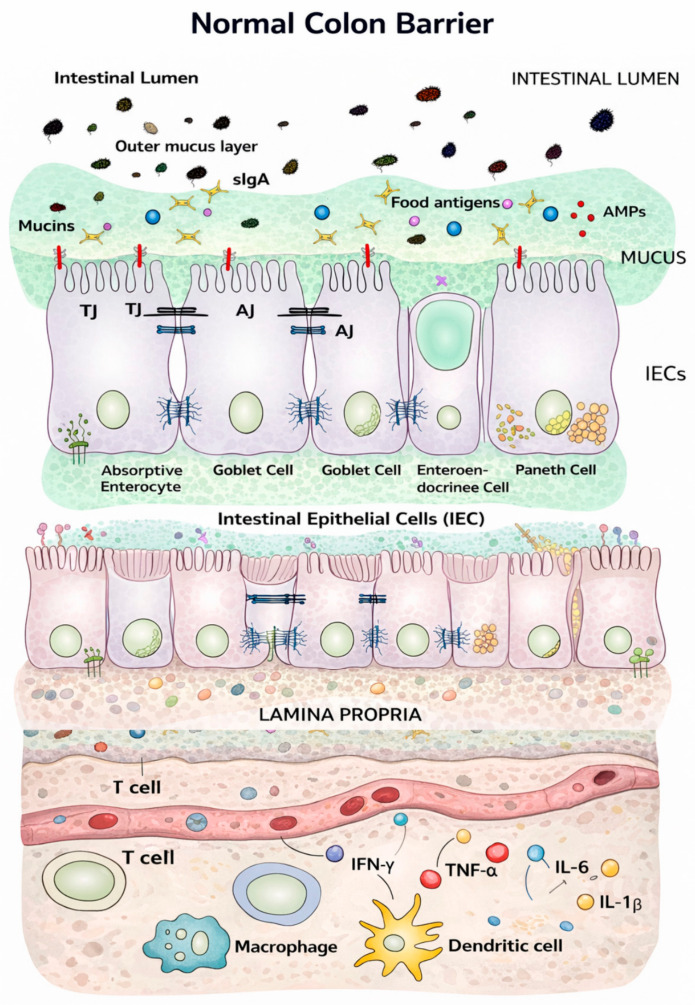
Normal epithelial barrier in the colon. Three layers of the colon are displayed (mucus layer, intestinal epithelial layer, and lamina propria).

The colon barrier is a highly dynamic interface that separates the interior lumen from the lamina propria. The colon consists of multiple layers, which are the mucus layer located nearest to the lumen, followed by the intestinal epithelial cells (IECs), and the lamina propria, as shown in Figure 1 [1]. The outer mucus layer is a habitat for commensal gut microbiota, whereas the inner mucus layer is devoid of bacteria [2]. Beneath the outer mucus layer are the IECs. IECs comprise five different cell types, which are absorptive enterocytes, goblet cells, enteroendocrine cells, Paneth cells, and microfold cells. Antimicrobial peptides (AMPs), secretory IgA (sIgA), and junctions (tight junctions (TJs), adheren junctions (AJs), and desmosomes) are also found within the IECs [1]. Each cell type has a unique role in protecting the colon environment and maintaining the barrier. Enterocytes absorb ions, water, nutrients, and vitamins through the apical plasma membrane and transport them across the basolateral plasma membrane [3]. Goblet cells secrete mucin and establish a protective mucus layer in the colon [4], while enteroendocrine cells produce gut hormones that coordinate food digestion and absorption [5]. Paneth cells which are abundant in small intestine, are found only in proximal colon [6]. These cells produce antimicrobial products or ingest intestinal microorganisms to protect IECs [7]. Microfold cells are responsible for transporting antigens and pathogens from the luminal area to the sub-epithelium via transcytosis [8]. Similarly, sIgA protects the epithelium from microbes and shapes the gut microbiome [9,10]. sIgA in the outer mucus layer binds with bacterial antigens, anchoring them in the outer layer, neutralizing, and thereby preventing microbial translocation into the lamina propria [10]. Besides immune exclusion (microbe coating), sIgA alters the biofilm composition of gut microbiome by adhering beneficial microbes on the outer layer, and outcompeting the pathogens [11]. O-antigens and teichoic acids in pathogens like proteobacteria can be detected by sIgA to maintain low affinity in the colon. AMPs, on the other hand, exhibit an anti-inflammatory response by recruiting immune cells to repair damaged IECs [12]. For instance, AMPs such as LL-37 and human defensins (hBD-2, hBD-3) in IECs bind directly to neutrophils to release neutrophil extracellular traps (NETs) or secrete chemokines like IL-8 to recruit more neutrophils to eliminate the pathogens [12,13]. In addition, AMPs such as PR-39 and JH-3, protect the barrier by inhibiting the apoptosis of immune cells responsible in barrier repair maintaining the integrity [12]. Moreover, AMPs, like tilapia piscidin 4 (TP4), reprograms pro-inflammatory M1 macrophages into anti-inflammatory M2 phenotypes explaining the dual role of AMPs in mediating pro-inflammatory cytokines in maintaining barrier integrity rather than its disruption effect [12,14]. The common method both sIgA and AMP plays in maintaining barrier integrity is by mediating the gut microbiome [15,16].

Beneath the IEC layer lies the lamina propria, which contains immune cells responsible for protecting the colon barrier. Although these components provide a protective environment in the colon, barrier dysfunction still occurs in humans. One of the initial and major inflammatory protections provided by immune cells is the synthesis of cytokines to signal and repair intestinal wounds [17]. In this review, four main pro-inflammatory cytokines: Interferon gamma (IFN-γ), tumor necrosis factor alpha (TNF-α), interleukin 6 (IL-6), and interleukin 1 beta (IL-1β), are studied in relation to their mediation of intestinal barrier dysfunction. Each type of pro-inflammatory cytokine has a different role in triggering immune cells and increasing inflammation. While they are often studied individually, this review provides an overview of the entire inflammatory network that leads to barrier breakdown.

Currently, gastrointestinal inflammation is primarily considered significant when diagnosed as inflammatory bowel diseases (IBDs), colorectal cancer (CRC), or colitis. This review analyses the barrier that fails to function and examines how pro-inflammatory cytokines act in parallel to promote inflammation and exacerbate barrier breakdown. We predict cytokines as the core mediator of epithelial barrier dysfunction and suggest that managing these signals can be an opportunity for the treatment of barrier dysfunction and intestinal inflammation.

Pro-inflammatory cytokines exert a dual influence on the intestinal barrier, acting as mediators that can both induce dysfunction and trigger protective mechanism. Although IL-6 disrupts intestinal barrier dysfunction, it also plays a role in the expansion of intestinal stem cells (ISCs) to seal physical gaps in the mucosa in the later part of damage and protects IECs from apoptosis [18]. IL-6 activating STAT3 pathway expresses TJ proteins claudin-2 and claudin-3 which in turn promote epithelial integrity [19]. Similarly, low-level TNF-α promotes the proliferation of IECs via TNFR2 signaling causing cell migration to cover damaged areas [20].

Beyond these traditional pro-inflammatory markers, pleiotropic cytokines, such as IL-22, exhibit distinct pro- and anti-inflammatory properties. IL-22 binds to the IL-22R1 receptor on ISC to stimulate the proliferation and expansion of new IECs, effectively healing barrier dysfunction [21]. Furthermore, IL-22 stimulates transit-amplifying cells to differentiate into new IECs. To prevent the post-effect of barrier dysfunction which is the microbial translocation, IL-22 triggers the secretion of AMPs such as Reg3α, DEFB103 (β-defensin 3), DMBT1, and LCN2 (lipocalin 2), which neutralize harmful pathogens [22]. Similarly, cytokines such as IL-17A and IL-17F, enhance barrier integrity by regulating the TJ protein occludin, thereby reducing the permeability in IECs. Clinical evidence has demonstrated that IL-17 therapy has replenished barrier integrity in IBD cases [23,24]. While acknowledging these protective functions, this review focuses specifically on the mechanism by which pro-inflammatory cytokines mediate intestinal barrier dysfunction, rather than their reparative roles.

## 2. Mechanisms of Epithelial Barrier Dysfunction in Intestinal Diseases

Before studying the role of cytokines in causing epithelial barrier dysfunction, it is important to understand the general mechanisms underlying this condition. Barrier dysfunction is defined as the loss of the barrier’s semipermeable function, which normally regulates the passage of essential nutrients while blocking pathogenic bacteria. This loss potentially harms the IECs by allowing an uncontrolled flux of antigens which triggers immune cells in the lamina propria [1]. The intestinal epithelial barrier has always served as the first line of defense. Loss of this function in the colon has been linked to intestinal diseases such as IBS, IBDs, and celiac disease. For example, alterations in the expression and distribution of claudin (CLDN-2), -5, and -8 have resulted in discontinuous tight junctions and barrier dysfunction in active Crohn’s disease (CD), proving that barrier dysfunction is a key risk factor for IBDs [25,26].

### 2.1. Tight Junction Protein Remodeling and Altered Paracellular Permeability

Epithelial barrier dysfunction rises from remodeling of junctional proteins, leading to abnormal paracellular permeability. TJs form a continuous intercellular barrier between epithelial cells, sealing paracellular openings, restricting mucosal-to-serosal transport of harmful luminal contents [27,28]. During barrier dysfunction, TJs undergo specific remodeling. For example, transmembrane proteins such as occludin (OCLN), CLDNs, junctional adhesion molecules (JAMs), tricellulin, and cytoplasmic scaffolding proteins, including zonula occludens (ZO)-1, ZO-2, and ZO-3, are redistributed [29]. In IBDs, barrier dysfunction is marked by selective downregulation or mislocalization of sealing CLDNs (e.g., CLDN-1, -3, -4, and -5), redistribution or internalization of OCLN and ZO-1, and upregulation of pore-forming CLDNs, particularly CLDN-2 [30]. This alteration in TJ composition transforms the epithelial barrier from a high-resistance, selectively permeable structure into a leaky epithelium that allows uncontrolled paracellular flux of ions and small solutes.

However, these alterations are not uniform across intestinal diseases or locations in the colon. CLDN-2 upregulation in IBD is prominent in inflamed epithelium and correlates with disease severity, whereas OCLN and ZO-1 downregulation are also observed in non-inflamed tissue [31,32]. In contrast, celiac disease exhibits reversible TJ rearrangement largely driven by dietary antigen exposure, highlighting the regulatory nature of TJ disruption [33].

TJs are also known as dynamic structures subject to continuous turnover via endocytic trafficking pathways. Under pathological conditions, cytokines, pathogens, oxidative stress, and calcium depletion induce selective internalization of junctional proteins through clathrin-mediated endocytosis [34], caveolar pathways, and micropinocytosis [35,36]. Internalized TJ proteins are targeted for degradation or recycled to the plasma membrane, depending on the nature and duration of the stimulus [37]. Disruption of normal TJ protein trafficking patterns results in persistent junctional disassembly and barrier loss [38].

### 2.2. MLCK Activation and Cytoskeletal Contraction

MLCK and Rho/ROCK signaling pathways play central roles in regulating these processes by modulating actin dynamics and vesicular transport [39]. Moreover, the apical junctional complex is mechanically coupled to the perijunctional actomyosin ring, making epithelial permeability highly sensitive to cytoskeletal tension [40]. The most common mechanism involved in cytoskeletal contraction is activation of myosin light chain kinase (MLCK) which induces phosphorylation of myosin light chains, leading to actomyosin contraction, junctional dilation, and increased paracellular permeability [41]. MLCK-mediated barrier dysfunction is size-selective, predominantly affecting permeability to small solutes, and occurs independently of epithelial apoptosis or structural damage [42]. Sustained MLCK activation is observed in chronic inflammatory states, including IBD and irritable bowel syndrome (IBS), causing low-grade barrier leakiness [43,44]. Experimental models show that epithelial-specific MLCK activation is sufficient to recruit immune cells and induce colitis, underscoring its causal role in disease initiation and progression [45].

### 2.3. Luminal Factors and Microbial Interactions

Furthermore, epithelial barrier dysfunction is caused by luminal factors and gut microbial interactions with junctional proteins. Pathogenic bacteria, including enteropathogenic and enterohemorrhagic *Escherichia coli*, *Campylobacter jejuni*, and *Clostridium difficile*, directly target junctional complexes by inducing dephosphorylation, internalization, or degradation of TJ proteins [46,47,48,49,50]. These effects are mediated through activation of protein kinase C, mitogen-activated protein kinase (MAPK), extracellular signal-regulated kinase (ERK), MLCK, and nuclear factor kappa-light-chain-enhancer of activated B cell (NF-κB) pathways which are often accompanied by cytoskeletal rearrangement [51]. In addition, bacteria-derived lipopolysaccharide (LPS) disrupts intestinal tight junction permeability in the colon by upregulating TLR-4 and CD14 expression [52]. High serum LPS levels in patients with type 2 diabetes indicate impaired intestinal barrier function [53].

Beyond pathogens and their byproducts, increased luminal protease activity contributes to barrier dysfunction, particularly in IBS and inflammatory conditions [54]. Protease-mediated activation of protease-activated receptor-2 (PAR-2) on epithelial cells triggers MLCK-dependent TJ opening and enhances visceral hypersensitivity [55]. For example, dietary gliadin peptides are found to stimulate ZO release via CXCR3 signaling, leading to reversible TJ disassembly and increased intestinal permeability in celiac disease [56].

### 2.4. Transcellular Antigen Uptake

Intestinal barrier dysfunction occurs due to transcellular antigen uptake [57]. Increased endocytosis and transcytosis of dietary antigens, macromolecules, and microbial products facilitate abnormal antigen presentation by enterocytes and promote mucosal immune activation [58,59]. For example, antigens are processed within endosomal compartments and presented via major histocompatibility complex molecules which activate adaptive immune responses in IBD and celiac disease [60,61]. This enhanced antigen trafficking is often associated with epithelial immaturity, cytoskeletal alterations, and disruption of brush border enzyme activity, contributing to malabsorption and sustained inflammation [62,63,64]. For example, food antigens such as oral collagen pass through the lamina propria and trigger the immune system to produce cytokines, activating TLR2 and TLR4 expression [65]. This, in turn, induces mucosal inflammation and intestinal barrier damage, affecting barrier function.

### 2.5. Genetic Susceptibility Causes Barrier Function

Barrier function is also compromised by genetic and systemic modulators. Genetic polymorphisms affect junctional scaffolding proteins and cytoskeletal regulators, such as MYO9B, PARD3, and MAGI2. Previous studies have shown that *MYO9B* gene polymorphisms cause ulcerative colitis (UC) and celiac disease [66] eventually leading to autoimmune disease [67]. Similarly, polymorphisms in MAGI2, which is involved in TJ assembly, are associated with Crohn’s disease and tumor suppression in several cancers. On the other hand, PARD3 encodes the protein PAR-3, which regulates epithelial cell polarity and facilitates tight junction formation [68]. Barrier genes such as PTGER4 and HNF4A, which encode proteins involved in junction redistribution and epithelial permeability, respectively, are highly upregulated in IBDs [69]. The *PTGER4* gene encodes EP4 receptors in IECs, which bind to prostaglandin E2 (PGE2) and are responsible for TJ disruption, inflammation, and immunomodulatory dysfunction [70,71]. The PGE2-EP4 complex promotes gut homeostasis by inducing anti-inflammatory IL-22 production [72], thereby restoring gut barrier function. Additionally, the *HNF4A* gene is a nuclear transcription factor involved in IECs’ cell division by transcribing TJ proteins [73,74]. Deletion of the *HNF4A* gene induces barrier dysfunction and inflammation [75].

Moreover, microRNA expression is strongly associated with barrier dysfunction and tight junction disassembly. In UC patients, *miR-192* is upregulated and *miR-16* is downregulated while in CD, *miR-23b* is upregulated and *miR-19bas* is downregulated; these changes correlate with diarrhea and barrier function [76]. Furthermore, the expression of each transmembrane protein is affected by microRNAs. For example, *miR-203*, *miR-483-3p*, and *miR-595* affect the expression of *ZO1* and *ZO2* transmembrane proteins. However, *miR-874* indirectly affects *CLDN-1* and is found to be inversely related to aquaporin 3 (*AQP3*) expression indicating high paracellular permeability. In IBD, *miR-122a* induces *OCLN* mRNA degradation, which contributes to barrier dysfunction by increasing permeability [76].

## 3. Role of TNF-α and IFN-γ in Junctional Disruption and Epithelial Apoptosis

**Figure 2:**
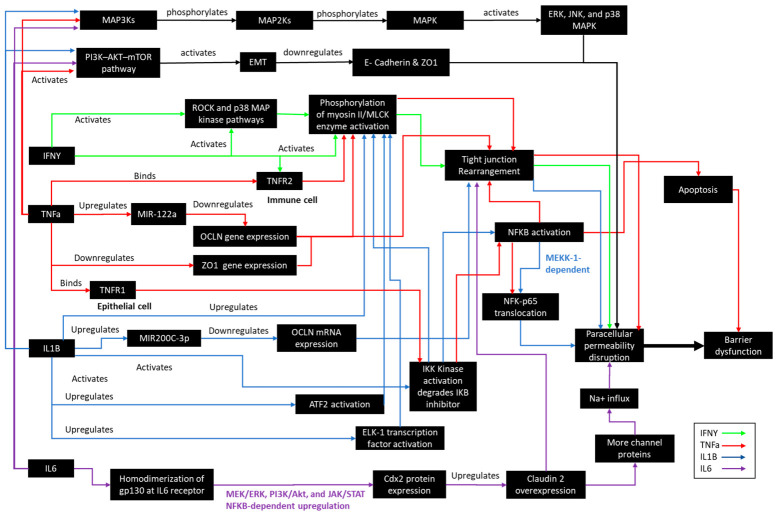
Sequential and synergistic cytokine-driven epithelial barrier dysfunction.

Among the factors that cause barrier dysfunction, cytokine signaling is a common contributor to alterations in the colon barrier, leading to an inflammatory state. Therefore, in this review, we focus on cytokine-mediated barrier dysfunction in colon. Two pro-inflammatory cytokines are highly associated with junctional disruption and epithelial apoptosis. Tumor necrosis factor alpha (TNF-α) is a potent inflammatory cytokine produced by macrophages that regulates inflammation, immune responses, cell division and proliferation, necrosis, and apoptosis, thereby creating resistance to cancerous infection [77,78]. In the early-inflamed colon, TNF-α rapidly induces the disassembly of TJs and causes epithelial cell death through upregulation of TNFR2 and the long isoform of myosin light chain kinase (MLCK) [34]. Activated MLCK then phosphorylates the myosin II light chain, causing the perijunctional actin-myosin ring to contract, which is pulling the TJs apart and widening the junction [40]. This mechanism leads to endocytosis of the TJ proteins, OCLN, and CLDN-2, disassembling them from the membrane, and creating cation-selective pores [34]. Depletion of OCLN by TNF-α involves upregulation of miR-122a (Figure 2) [76]. CLDN-2 is a pore-forming protein that allows water and ions to leak through the barrier [79]. These events abnormally open the paracellular leak pathway in the colon epithelium, disrupting the barrier function.

As noted in early inflammation models, barrier breakdown is measured by increased permeability through upregulation of TNFR2 and MLCK in IECs [80]. However, as inflammation progresses, TNF-α also binds to TNFR1 in IECs, which assembles the FADD and procaspase-8 containing complex II [81]. The activation of caspase-8 then triggers caspase-3-mediated apoptosis in epithelial cells [82]. Caspases are endoproteases that regulate cellular networks, controlling inflammation and cell death [83]. Therefore, activation of caspases in the colon supports the role of TNF-α in inducing apoptosis in IECs, leading to barrier dysfunction [84]. If the redistribution of junctional proteins in adjacent cells fails to close the gap, transient leaks occur, indicating barrier breakdown.

IFN-γ is a pro-inflammatory cytokine encoded by the *IFNG* gene that regulates immune and cellular responses and initiates downstream signal transduction cascades, responsible for gene expression regulation [85]. There are type I, type II, and type III IFN, all of which share the ability to promote antiviral activities activated by interaction with the IFN-γR receptor [86,87]. During intestinal diseases, IFN-γ induces macropinocytosis, causing the apical membrane to fold inward and engulf junctional proteins (e.g., OCLN, JAM-A, and CLDN-1) into large actin-coated vacuoles known as vacuolar apical compartments (VACs) [35]. Although IFN-γ redistributes TJ proteins specifically via micropinocytosis, both TNF-α and IFN-γ redistribute TJ proteins via endocytosis.

Unlike TNF-α, IFN-γ reduces rather than redistributes junctional proteins. Despite this protein loss, IFN-γ activates Fyn kinase, leading to the internalization of E-cadherin and subsequent disruption of TJ structure [88]. For example, in human colonic T84 epithelial cells, IFN-γ stimulation induces Src-family kinase-dependent tyrosine phosphorylation of E-cadherin and its stabilizing unit p120-catenin [88]. This results in the disassembly of the AJ complex from the plasma membrane, followed by the redistribution of E-cadherin, p120-catenin, and β-catenin from an insoluble membrane-associated complex into free soluble cytoplasmic molecules. Redistribution of junctional proteins disrupts the stable cell–cell adhesion, resulting in barrier breakdown. Furthermore, phosphorylation of E-cadherin is detected by the E3 ubiquitin ligase Hakai, which ubiquitinates and degrades E-cadherin [89]. This weakens the intercellular adhesion, leaving the epithelial monolayer mechanically fragile. These findings demonstrate that IFN-γ affects epithelial barrier integrity not only through TJ distribution but also via AJ disassembly and by E-Cadherin degradation, leading to barrier breakdown during intestinal inflammation.

Moreover, IFN-γ enhances the effects of TNF-α by inducing epithelial TNF receptor 2 expression via STAT 1 signaling, sensitizing cells to TNF-driven MLCK activation [90]. In previous studies, IFN-γ was even used as TNF-α pre-treatment to elevate MLCK expression and disrupt barrier function [91]. The difference between IFN-γ and TNF-α is that TNF-α triggers apoptosis and cell shedding along with junctional disassembly, whereas IFN-γ alone displaces selected TJ proteins without causing apoptosis [92]. Although IFN-γ and TNF-α act differently to regulate the inflamed or diseased colon, both cytokines are interdependent and lead to barrier dysfunction resulting in co-mediation.

## 4. Role of IL-1β and IL-6 in Permeability Amplification and Sustained Inflammation in the Colon

IL-1β and IL-6 amplify barrier leak and promote chronic inflammation through different but overlapping mechanisms. IL-1β significantly increases colonic paracellular permeability by activating the NF-κB signaling pathway [93]. In IECs, IL-1β degrades inhibitor of kappa B alpha (IκBα), which maintains the deactivation of the NF-κB transcription factor in the cytoplasm. Activation of the NF-κB pathway upregulates inflammatory genes [94] and enables nuclear translocation of NF-κB p65/p50, which upregulates MLCK transcription [94]. Upregulation of MLCK-driven actomyosin contraction mechanically widens the TJ, increasing permeability [40]. This is how IL-1β amplifies permeability, indicating barrier dysfunction.

In addition, IL-1β downregulates OCLN by upregulating *microRNA-200c-3p* gene expression, weakening the apical junctions (Figure 2) [95] and modulating inflammation [94]. OCLN, which is crucial for CK2-mediated barrier regulation upon phosphorylation at S408, stabilizes tight junction strand organization and epithelial barrier function by reducing paracellular permeability [96]. This proves that IL-1β is responsible for opening the leak pathway of the colonic barrier through the NF-κB pathway, MLCK activation, and destabilization of OCLN junctions. These mechanisms led by IL-1β may be slower compared to TNF-α-induced TJ redistribution, which uses the TNFR2/MLCK trigger; however, IL-1β can sustain permeability even in the absence of overt cell death [97]. Moreover, the slower barrier breakdown signaled by IL-1β may be due to transcriptional and post-transcriptional mechanisms, including NF-κB-dependent induction of MLCK and microRNA-mediated suppression of OCLN expression.

Unlike IL-1β, TNF-α, and IFN-γ, which target ZO and OCLN, IL-6 amplifies gut permeability by targeting CLDN. IL-6 upregulates the expression of CLDN-2, a cation-selective channel protein, through the c-Jun N-terminal kinase (JNK/AP-1) pathway [98]. IL-6 causes small ions (<4 Å) to leak through the paracellular route via newly expressed CLDN-2 pores [98]. This JNK/AP-1-CLDN-2 mechanism has been demonstrated in colonic epithelial monolayers and mouse intestines, showing that IL-6 can widen the TJ to enable ion flow. Notably in IECs, IL-6 signals through both classic IL-6R (STAT3) and trans-signaling modes, but the permeability effect arises from the kinase cascade and transcription factor AP-1 [99]. IL-6 homodimerizes gp130 at the IL-6 receptor to upregulate Cdx2 protein expression. Cdx2 protein expression leads to CLDN-2 overexpression, which enables sodium ion influx into IECs [100].

Together, IL-1β and IL-6 amplify each other’s effects in a feed-forward loop, increasing permeability and sustaining inflammation in the colon. IL-1β stimulation of IECs drives de novo IL-6 production via NF-κB [101]. Moreover, both IL-1β and IL-6 promote Th17 polarization by inducing naive T cells to become IL-17-secreting Th17 cells [102]. IL-17 then recruits neutrophils and macrophages, which sustain inflammation. In return, neutrophils and macrophages produce and mature IL-1β, which can maintain or even increase inflammation, leading to IBDs and colitis. Meanwhile, IL-6/STAT3 signaling promotes the survival of inflammatory T cells by regulating Bcl-2/Bcl-xL, preventing apoptosis of Th1/Th17 cells in the mucosa [102]. Therefore, IL-6 not only increases epithelial permeability but also preserves the immune response. In summary, IL-1β acutely disrupts the barrier via MLCK/NF-κB, while IL-6 reinforces this leak (via CLDN-2) and sustains chronic inflammation by supporting Th17/Th1 cell survival [101,102].

## 5. Cytokine Synergy and Chronicity: Why Damage Persists

Pro-inflammatory cytokines act as highly interconnected mediators, forming self-reinforcing networks in the human colon. For instance, IFN-γ produced by Th1 cells activates macrophages and dendritic cells to release TNF-α, IL-1β, and IL-6, initiating a coordinated inflammatory cascade [103]. Activated macrophages and dendritic cells sustain the response by releasing these same cytokines, ensuring prolonged inflammatory signaling. This causes intracellular pathways such as NF-κB, JAK–STAT, and MAP kinase cascades to remain active. IL-6 and TNF-α together drive STAT3 and NF-κB in immune cells to amplify cytokine gene transcription [104], while IL-1β and TNF-α signals are activated by priming each other’s receptor systems [105], including IFN-γ–induced upregulation of TNFR2 in IECs [106].

In some cases, three of the pro-inflammatory cytokines TNF-α, IL-1β, and IL-6 work together inducing barrier dysfunction. MAPK pathway represents a central signaling axis activated downstream of cytokine–receptor interactions at the IEC membrane [107]. TNF-α, IL-1β, and IL-6 initiate MAPK signaling by binding to their respective transmembrane receptors, triggering a cascade of phosphorylation events that culminate in the activation of key MAPK subfamilies ERK, JNK, and p38 MAPK [108]. Pro-inflammatory cytokine binding recruits TNF receptor-associated factors (TRAFs) and myeloid differentiation primary response protein 88 (MyD88) [109]. These molecules assemble signaling complexes and bridge receptor activation to downstream kinase cascades, where MAPK kinases MAP3Ks [110], activated MAP3Ks phosphorylate, and activate MAP2Ks, such as MEK1/2, MKK4/7, and MKK3/6. Subsequently, these MAP2Ks phosphorylate MAPKs, activating the MAPK pathway, which in turn induces barrier dysfunction in colon [111]. MAPK signaling is also tightly integrated with immune system function. It regulates key processes such as macrophage activation, T cell differentiation, and the production of pro-inflammatory cytokines [112].

The phosphatidylinositol 3-kinase (PI3K)–AKT–mechanistic target of rapamycin (mTOR) signaling pathway plays a central role in regulating intestinal epithelial integrity, immune responses, and cellular metabolism [113]. In the colon, pro-inflammatory cytokines such as TNF-α, IL-1β, and IL-6 activate this pathway through membrane receptor-mediated mechanisms. When pro-inflammatory cytokines bind to their respective receptors on IECs and immune cells, PI3Kβ isoform is activated and vice versa [114]. Activated PI3Kβ converts phosphatidylinositol 4,5-bisphosphate (PIP2) into phosphatidylinositol 3,4,5-triphosphate (PIP3), which recruits and activates AKT [113]. Activated AKT phosphorylates mTOR. Activation of PI3K–AKT–mTOR pathway induces epithelial–mesenchymal transition (EMT) and disrupts the cytoskeletal structure [115]. During EMT, epithelial cells that maintain tight functional barriers dysfunction as they acquire mesenchymal phenotype. This transition downregulates E-cadherin and ZO-1 [116]. The loss of these junctional proteins weakens intercellular connections, compromising barrier function. PI3K–AKT–mTOR pathway also directly regulates cytoskeletal organization through activation of mTORC1 and mTORC2 complexes [115]. These complexes modulate the dynamics of structural components such as F-actin microfilaments and β-tubulin microtubules. As a result, the structural framework required to anchor junctional proteins is destabilized, thereby weakening epithelial cohesion. Therefore, activation of PI3K–AKT–mTOR pathway might disrupt the epithelial barrier. Both PI3K–AKT–mTOR and MAPK pathways are activated by pro-inflammatory cytokines and are also being drivers to produce them in IECs.

Figure 2 shows the interconnected cascade of pro-inflammatory cytokine-mediated epithelial barrier dysfunction. Phosphorylation of myosin II through activation of the MLCK enzyme is the primary mechanism shared by pro-inflammatory cytokines in redistributing TJs and inducing barrier dysfunction. However, IL-6 redistributes TJs in IECs by overexpressing CLDN-2 and increasing channel proteins, which directly raises paracellular permeability without involving MLCK activation. The ultimate effect is the same as with other cytokines, but the pathway differs. In addition to MLCK activation, pro-inflammatory cytokines also act synergistically in regulating transmembrane protein where *OCLN* gene downregulation and OCLN mRNA downregulation are carried out by TNF-α and IL-1β respectively, while *ZO1* gene downregulation and *CLDN-2* gene overexpression are mediated by TNF-α and IL-6, respectively. All cytokines activate the NF-KB pathway via tight junction disassembly, but TNF-α and IL-1β have a distinct route by activating IKK kinase which degrades the IKB inhibitor. Degradation of the IKB inhibitor leads to translocation of NF-κB p65 which then activates NF-KB pathway. NF-KB activation by TNF-α causes apoptosis of IECs whereas IL-1β increases paracellular permeability. Among these pro-inflammatory cytokines, TNF-α is the most impactful driver of colon barrier disruption due to its apoptotic mechanism. Based on Figure 2, IL-1β and TNF-α share more mechanisms in barrier dysfunction, while IL-6 primarily targets a specific transmembrane protein (CLDN-2). IFN-γ act as the initiator, providing the foundation for the cascade, especially for the TNF-α-mediated pathway. Upregulation of TNFR1 and TNFR2 expression in IECs increases the likelihood of TNF-α binding to its receptors.

Based on the interconnected cascade, four phases can be identified in pro-inflammatory cytokine-mediated barrier dysfunction. The first is the initiation phase, in which IFN-γ activates TNF-α pathways. Next, TNF-α and IL-1β together increase cytoskeletal tension, resulting in tight junction opening and early paracellular permeability. TNF-α and IL-1β act as transcriptional sustainers, maintaining prolonged inflammatory signaling within epithelial cells and promoting continued cytokine production. As inflammation progresses, TNF-α, as the primary damage-causing cytokine, drives the apoptotic and epithelial shedding phase, where epithelial cell loss causes irreversible disruption of the monolayer. Finally, in the amplification phase IL-6 as a dysfunction amplifier induces chronic paracellular permeability through sustained channel pore formation and immune activation, ensuring continuous barrier leak even in the absence of acute epithelial death. Persistent barrier dysfunction is strongly associated with the cyclic nature of the pro-inflammatory cytokine-mediated mechanism. Triggered immune cells produce cytokines that drive barrier dysfunction, and barrier dysfunction, in return, stimulates immune cells to continually produce cytokines, sustaining damage to the epithelial barrier.

In current therapeutics, neutralizing a single cytokine does not fully restore barrier integrity. For example, TNF-α blockade does not prevent barrier breakdown because parallel inflammatory pathways, driven by IL-1β and IL-6, remain active [117]. Among these cytokines, IL-6 plays a pivotal role in converting transient epithelial injury into chronic inflammation by sustaining Th1 and Th17 immune responses [102]. The cumulative effect is a stable pathological state in which epithelial repair is continuously opposed by overlapping cytokine signals [118]. Histologically, barrier breakdown is characterized by chronic tight junction remodeling, persistent immune-cell infiltration, and failure of the epithelial barrier to reseal [119,120]. Multiple cytokines converge on shared molecular axes, locking the mucosa into a feed-forward inflammatory loop that disrupts the barrier. As barrier loss persists, permeability increases through both the pore and leak pathways because the junctional proteins in IECs are the components most affected by pro-inflammatory cytokines [121]. This allows more bacteria and antigens to bypass the mucin and IEC layer, gaining access to the lamina propria. In the lamina propria, increased immune-cell activity perpetuates pro-inflammatory cytokine release, further enhancing barrier breakdown [122].

There are two signaling pathways involved in this pro-inflammatory cytokine-based barrier dysfunction interconnected cascade. The initial stage starts with immune signaling followed by epithelial signaling. The production of immune-cells and cytokines occurs via immune cell signaling while the cytokine receptor, binding leading barrier integrity disruption, involves epithelial signaling.

## 6. Therapeutic Targeting of Cytokine Networks to Restore Barrier Integrity

Over the years, therapeutics targeting cytokine networks to reduce inflammation and restore barrier integrity have advanced significantly. There are two types of therapeutics involving cytokines. Based on Table 1, the most common approach is neutralizing pro-inflammatory cytokines, using such as Infliximab, Adalimumab, and Golimumab, which inhibit the TNF-α pathway [123]. These drugs prevent MLCK activation, stabilize OCLN, CLDN-1, and ZO-1, and reduce epithelial apoptosis. However, the IL-1β and IL-6 pathways often remain active. Anakinra and Canakinumab target IL-1β. By preventing TJ destabilization, OCLN phosphorylation, and endocytosis, these drugs prevent epithelial barrier dysfunction. This mechanism is not efficient in mild inflammation due to its slow onset. Moreover, Tocilizumab and Sarilumab block IL-6 trans-signaling, preventing chronic permeability and Th17-driven inflammation. However, studies indicate a potential risk of impairing acute mucosal repair. For IFN-γ, experiments prove Fontolizumab reduces CLDN-2 upregulation and limits epithelial apoptosis and antigen transcytosis.

Anti-inflammatory cytokine enhancement is the second type of therapeutic approach involving cytokines. Anti-inflammatory cytokines such as IL-4, IL-10, IL-11, and IL-13 are immunoregulatory molecules that control the pro-inflammatory cytokine response, primarily by inhibiting the receptors [147]. Therefore, enhancing anti-inflammatory cytokines is an indirect method to modulate pro-inflammatory cytokines and prevent barrier dysfunction. Recombinant IL-10 and IL-10-inducing probiotics are used as therapeutics to counteract TNF-α which promotes TJ reassembly and immune tolerance. However, efficacy in humans is inconsistent. To enhance epithelial restitution and wound healing, TGF-β is used to treat barrier dysfunction, but there is a risk of fibrosis if dysregulated.

Besides cytokine-mediated therapeutics, there are signaling hub inhibitors, TJ-focused therapies, microbiota-based modulation, and regenerative approaches. Each cytokine is involved in specific signaling hubs such as the JAK–STAT, NF-κB, and MAPK/p38 pathways. Inhibiting these pathways is one way to treat barrier dysfunction. Tofacitinib and Upadacitinib are used to inhibit the JAK-STAT pathway by restoring TEER and suppressing MLCK. However, an increased risk of infection is observed with long-term use of these drugs. To inhibit the NF-κB pathway, corticosteroids, prednisone, and IKK inhibitors are used, although they are non-specific and have poor long-term safety profiles. For MAPK/p38 inhibition, there are no FDA-approved drugs, and experimental inhibitors are still under study. Although, in theory MAPK/p38 inhibition prevents cytokine-induced TJ phosphorylation and internalization, most preclinical candidates have been withdrawn due to side effects. This indicates that controlling multiple cytokine-mediated signaling pathways still needs improvement, unlike single cytokine therapeutics.

TJ-focused therapy is also used to treat barrier dysfunction, although clinical translation remains limited. SAR442970 and anti-CLDN 2 agents, which are currently in a Phase 2b clinical trial, are used as a pore-closing therapy. Similarly, Larazotide Acetate, in a Phase III Pivotal trial, has been shown to prevent zonulin-induced rearrangement of CLDNs. Experimental inhibitors of MLCK that prevent actomyosin contraction and preserve paracellular sealing are still under investigation. Barrier integrity can also be protected and treated through gut microbiome modulation. Cytokines can be modulated indirectly via alterations in the colonic microbiota. Fecal microbiota transplantation increases anti-inflammatory cytokines IL-25 and IL-10, while decreasing Th17 cells. Some therapies focus on balancing Firmicutes/Bacteriodetes ratio. For example, Vowst (SER-109) and Rebyota (RBX2660) are used to produce secondary bile acids, which have direct anti-inflammatory effects, e.g., IL-6 suppression. A limitation of microbiota-based modulation is that treatment depends on strain- and host-specific effects.

Regenerative approaches include growth factor signaling such as EGFR and GLP-2. EGF, as studied in clinical trials, inhibits the TNF-α-mediated pathway to treat barrier dysfunction, while GLP-2 increases the production of IGF-1, which protects cells from apoptosis. However, these signals could promote continuous cell growth, potentially leading to uncontrolled proliferation. In addition to growth factors, mesenchymal stem cells are used in MSC therapy to secrete immunosuppressive molecules (e.g., IDO, TGF-β, and IL-10), shifting macrophages to an anti-inflammatory M2 phenotype. MSC therapy is expensive. Emerging treatments for barrier dysfunction also focus on genetic modulation using CRISPR technology to reprogram cells to resist pro-inflammatory cytokines, inhibiting harmful pathways while preserving beneficial signals. However, ethical and safety measures need to be considered in this therapy.

## 7. Rationale for Multi-Cytokine or Pathway-Based Interventions

The main limitation of single-cytokine therapies is that pro-inflammatory cytokines do not act independently. Barrier dysfunction is more severe due to the synergistic, rather than additive, interaction of multiple cytokines. This provides a strong mechanistic rationale for multi-cytokine targeting strategies. By simultaneously neutralizing synergistic cytokine pairs, the multiplier effect of pro-inflammatory cytokines can be disrupted. TJ failure can be prevented by levels of mucosal healing that single-agent therapy cannot consistently provide.

Despite the clinical achievements of biologics, long-term data indicate an unbreakable therapeutic ceiling. Approximately 30–50% of patients achieve sustained mucosal healing biologic therapy [148]. This plateau reflects the redundant architecture of inflammatory signaling networks rather than drug inefficacy.

When TNF-α is inhibited, IL-1β and IL-6 can activate overlapping transcriptional programs that trigger barrier-disruptive pathways. Therefore, the inflammatory pathway is replaced but not completely inhibited by the current therapeutics. This limitation provides a rationale for targeting central signaling hubs, such as the JAK–STAT pathway. JAK inhibitors (e.g., upadacitinib) do not simply block individual cytokines but instead shut down the shared intracellular process [149]. By simultaneously attenuating signaling downstream of IL-6, IFN-γ, IL-12, and IL-23, these agents prevent the immune system from rerouting inflammatory activity through compensatory pathways, thereby offering a more durable restoration of barrier integrity. Effective restoration of the colonic barrier requires two biologically distinct processes: (1) immediate sealing of paracellular leaks and (2) long-term regeneration of the epithelial lining. Most conventional anti-inflammatory therapies excel at the former but are largely ineffective at the latter [150].

While cytokine suppression can stabilize TJs and reduce permeability, chronic inflammatory signaling—particularly through IL-23-driven immune activation—impairs intestinal stem cell survival and differentiation [151]. Consequently, the repair mechanism within epithelial cells remains functionally suppressed even after inflammation is reduced since the damage affects the stem cells. This insight drives interest in combination and multi-pathway therapies, exemplified by strategies such as dual TNF-α and IL-23 blockade. In this framework, TNF inhibition prevents junctional disruption, while IL-23 inhibition relieves inflammatory pressure on LGR5^+^ intestinal stem cells, permitting effective epithelial renewal [152]. Such approaches stress that barrier restoration is not just an immunological outcome but a regenerative process that requires permissive signaling environments.

Once barrier integrity is compromised, luminal microbial products translocate into the mucosa, initiating escalating inflammation and progressive permeability [153,154]. Damaged epithelial cells release alarmins, such as IL-33 and TSLP, which serve as upstream danger signals that recruit and activate multiple immune-cell populations [155]. These alarmins trigger broad cytokine cascades, including TNF-α, IL-1β, IL-6, and IFN-γ [156]. Moreover, microbial translocation increases systemic pro-inflammatory cytokines IFN-α, IL-1, IL-6, IL-18, and TNF-α, as well as the anti-inflammatory cytokine TGF-β, simultaneously activating innate immune cells and promoting CD4 T cell restoration [157]. This shows that blocking aggressive downstream cytokines while neglecting upstream epithelial danger signaling is a poor therapeutic approach. Targeting these early alarm pathways could serve as a source-control strategy, aiming to extinguish inflammation before it diversifies into a multi-cytokine storm. Therefore, multi-cytokine or pathway-mediated therapeutics could represent a breakthrough in treating colon barrier breakdown by suppressing all related cascades, including inflammation and increased permeability.

## 8. Conclusions

Pro-inflammatory cytokines mediate colon barrier breakdown through NF-κB-driven inflammation and MLCK regulation, JAK/STAT-mediated immune-cell survival, MAP kinase-dependent transcriptional control, and caspase-mediated epithelial apoptosis, causing tight junction opening, protein mislocalization, and epithelial cell loss. Pro-inflammatory cytokines interact within a very highly connected network, triggering inflammation and resulting in leaky gut and barrier dysfunction. The most commonly shared mechanism is the tight junction rearrangement which directly affects the permeability function of the barrier. Further treatment studies are needed to identify drugs that can treat inflammation and barrier dysfunction by inhibiting overall pro-inflammatory cytokine mechanisms rather than targeting individual cytokines.

## Figures and Tables

**Table 1:** Therapeutic strategies targeting cytokine-driven epithelial barrier dysfunction.

Therapeutic Strategy	Target Cytokine/Pathway	Representative Agents/Approaches	Key Mechanisms on Barrier Integrity	Key Limitations	References
Cytokine Neutralization	TNF-α	Infliximab, Adalimumab, Golimumab	Prevents MLCK activationStabilizes OCLN, CLDN-1, ZO-1 Reduces epithelial apoptosis;	IL-1β and IL-6 pathways often remain active	[123,124,125]
	IL-1β	Anakinra, Canakinumab	inhibits sustained TJ destabilization Reduces OCLN phosphorylation and endocytosis;	Slower onset; mainly effective in chronic inflammation	[126,127]
	IL-6	Tocilizumab, Sarilumab	blocks IL-6 trans-signaling; prevents chronic permeability and Th17-driven inflammation	May impair acute mucosal repair	[128,129]
	IFN-γ	Fontolizumab (experimental)	Reduces CLDN-2 upregulation; limits epithelial apoptosis and antigen transcytosis	Limited clinical availability	[130]
Signaling Hub Inhibition	JAK–STAT	Tofacitinib, Upadacitinib	Simultaneous inhibition of TNF-α, IL-6, IFN-γ signaling; restores TEER; suppresses MLCK	Infection risk with long-term use	[131,132]
	NF-κB	Corticosteroids, Prednisone, IKK inhibitors	Broad suppression of pro-inflammatory cytokine transcription; reduces epithelial apoptosis	Non-specific; poor long-term safety	[133]
	MAPK/p38	No FDA-approved drugs Experimental inhibitors under study	Prevents cytokine-induced TJ phosphorylation and internalization	Mostly preclinical and withdrawn due to side effects	[134]
TJ-Focused Therapy	MLCK	No FDA-approved drugs Experimental inhibitors under study	Prevents actomyosin contraction and preserves paracellular sealing	Limited clinical translation	[40]
	CLDN modulation	SAR442970 (Phase 2b clinical trial)	Anti-CLDN-2 (pore closing therapy)	Limited clinical translation	[135]
		Larazotide Acetate (Phase III Pivotal trial)	prevents the zonulin-induced rearrangement of CLDNs	Limited clinical translation	[136]
Anti-Inflammatory Cytokine Enhancement	IL-10	Recombinant IL-10, IL-10-inducing probiotics	Counteracts TNF- α and promotes TJ reassembly and immune tolerance	Variable efficacy in humans	[137]
	TGF-β	Endogenous pathway activation	Enhances epithelial restitution and wound healing;	Fibrosis risk if dysregulated	[138]
Microbiota-Based Modulation	Cytokine balance via microbiota	Fecal microbiota transplantation	increase anti-inflammatory cytokines IL-25 and IL-10, while decreasing the Th17 cells	Strain- and host-dependent effects	[139,140]
	Firmicutes/Bacteriodetes ratio balance	Vowst (SER-109) Rebyota (RBX2660)	Produces secondary bile acids, which have direct anti-inflammatory effects IL-6 suppression	Strain- and host-dependent effects	[141,142]
Regenerative Therapies	Growth factor signaling	Epidermal growth factor (EGF) (clinical trials) GLP-2	EGFR inhibits the TNF-α-mediated pathway GLP-2 increases the production of IGF-1, which protects cells from apoptosis	Signals could keep the cells grow (uncontrolled growth)	[143,144]
	Mesenchymal stem cells	MSC therapy	Secrete immunosuppressive molecules like IDO, TGF-β, and IL-10, shifting macrophages to an anti-inflammatory M2 phenotype	Cost and scalability issues	[145]
Emerging Strategies	Genetic modulation	CRISPR-based approaches	Reprograms the cells to resist cytokines while preserving good signals Inhibit NF-κB pathway	Ethical and safety measures	[146]

## Data Availability

No new data were created or analyzed in this study. Data sharing is not applicable to this article.

## Abbreviations

The following abbreviations are used in this manuscript:

AMP	Antimicrobial peptides
FDA	Food and Drug Administration
TJ	Tight junction
TNF-α	Tumor Necrosis Factor Alpha
IL-1β	Interleukin 1 Beta
IL-6	Interleukin 6
IFN-γ	Interferon gamma
IFN	Interferon
TNFR	Tumor Necrosis Factor Receptor
MLCK	Myosin light chain kinase
MAP	Mitogen-activated protein
IEC	Intestinal epithelial cell
ISC	Intestinal stem cell
OCLN	OCLN
CLDN	CLDN
IBD	Inflammatory Bowel Disease
CRC	Colorectal cancer
CD	Crohn’s disease
UC	Ulcerative colitis
ZO	Zonula occludin
EGF	Epidermal growth factor
EGF	Epidermal growth factor receptor
NF-κB	Nuclear Factor kappa-light-chain-enhancer of activated B cells
